# The Implication of Autophagy in Gastric Cancer Progression

**DOI:** 10.3390/life11121304

**Published:** 2021-11-27

**Authors:** Evangelos Koustas, Eleni-Myrto Trifylli, Panagiotis Sarantis, Nikolaos I. Kontolatis, Christos Damaskos, Nikolaos Garmpis, Christos Vallilas, Anna Garmpi, Athanasios G. Papavassiliou, Michalis V. Karamouzis

**Affiliations:** 1 Molecular Oncology Unit, Department of Biological Chemistry, Medical School, National and Kapodistrian University of Athens, 11527 Athens, Greece; trif.lena@gmail.com (E.-M.T.); panayotissarantis@gmail.com (P.S.); nick.kon3@gmail.com (N.I.K.); chris-vallilas@hotmail.com (C.V.); papavas@med.uoa.gr (A.G.P.); mkaramouz@med.uoa.gr (M.V.K.); 2 Renal Transplantation Unit, ‘Laiko’ General Hospital, 11527 Athens, Greece; x_damaskos@yahoo.gr; 3 ‘N.S. Christeas’ Laboratory of Experimental Surgery and Surgical Research, Medical School, National and Kapodistrian University of Athens, 11527 Athens, Greece; nikosg22@hotmail.com; 4 Second Department of Propedeutic Surgery, ‘Laiko’ General Hospital, Medical School, National and Kapodistrian University of Athens, 11527 Athens, Greece; 5 First Department of Pathology, Medical School, National and Kapodistrian University of Athens, 11527 Athens, Greece; annagar@windowslive.com

**Keywords:** autophagy, autophagy inducers, autophagy inhibitors, autophagy regulation, chemotherapy, gastric cancer

## Abstract

Gastric cancer is the fifth most common malignancy and the third leading cause of cancer-related death worldwide. The three entirely variable entities have distinct epidemiology, molecular characteristics, prognosis, and strategies for clinical management. However, many gastric tumors appear to be resistant to current chemotherapeutic agents. Moreover, a significant number of gastric cancer patients, with a lack of optimal treatment strategies, have reduced survival. In recent years, multiple research data have highlighted the importance of autophagy, an essential catabolic process of cytoplasmic component digestion, in cancer. The role of autophagy as a tumor suppressor or tumor promoter mechanism remains controversial. The multistep nature of the autophagy process offers a wide array of targetable points for designing novel chemotherapeutic strategies. The purpose of this review is to summarize the current knowledge regarding the interplay between gastric cancer development and the autophagy process and decipher the role of autophagy in this kind of cancer. A plethora of different agents that direct or indirect target autophagy may be a novel therapeutic approach for gastric cancer patients.

## 1. Introduction

Gastric cancer (GC) constitutes the fourth most frequent cause of death, due to malignancy and the fifth most commonly detected cancer worldwide [1]. A higher incidence is demonstrated in many countries among the continents such as in Western and Eastern Asia, Eastern Europe, and South America [2]. The gender disparity is reflected by the cumulative risk of mortality from birth till the age of 74 years, which is 0.57% for women and 1.36% for men. Despite the continuous amplification of GC cases in the last five decades, this trend is nowadays gradually decreasing due to the more efficacious treatment regimens for *Helicobacter pylori* (*H. pylori*) eradication, which composes a major factor for gastric carcinogenesis [3]. In view of the above, GC exhibits not only geographical variation, implying the influence of local environmental risk factors but also male predominance, with two-fold higher incidence for men [4,5], whereas the risk is equal for post-menopause women [6]. A familial predisposition for GC is demonstrated in the minority of GC cases (10%), while 1–3% of them are correlated with inherited syndromes such as gastric adenocarcinoma and proximal polyposis of the stomach syndrome (GAPPS), diffuse gastric cancer (HDGC), familial adenomatous polyposis (FAP), andhereditary non-polyposis colorectal cancer (HNPCC), and PeutzJegher’s syndrome [7]. 

The subdivision of GC is anatomically based, with two entities: the (i) non-cardia GC and the ii) cardia GC. The former is reported twice asfrequently asthe latter [8], constituting the majority of the cases (80–90%), and it is associated with *H. pylori* infection [9], as well as with dietary habits [10], economical, and sociological state, while the latter has an epidemiological background resembling that of esophageal adenocarcinoma (EAC), mostly in developed countries [11]. Different risk factors are taking part in gastric carcinogenesis based on the anatomical region. A stepping stone in distal, mainly antral, non-cardia GCs is *H. pylori*. Infection, resulting in gastritis and ulcers formation [12], increases almost six-fold the risk for GC in chronic infection in a span of ten years [13]. Based on AGA- 2020 Clinical practice guidelines, recommendation 1, patients with positive biopsies for pre-dysplasia stages asin gastric intestinal metaplasia (GIM) must be tested for *H. pylori*, and if infection occurs, it must be eradicated [14], which significantly reduces the risk for GC [15]. Obesity is linked with cardia GC, while esophageal pathologies such asBarrett’s esophagus and gastroesophageal reflux disease (GERD) are correlated with carcinogenesis in gastroesophageal junction [16]. Viral infection with EBV increases the risk of cancer development [17], while it accounts the 10% of the intestinal entity of GC, related also with microsatellite instability (MSI) [18,19]. Iatrogenic risk factors promote gastric carcinogenesis, such asthe long-term abuse of proton-pump inhibitors(PPIs) [19] and Bill Roth anastomosis [20,21]. 

Gastric carcinogenesis is a multifactorial event arising from deregulated pathways of signaling, mutated genes, and epigenetic aberrations, in combination with the influence of environmental factors.A huge range of natural products including tunicamycin, medicinal plants and microorganisms including flavonoids, coumarins, terpenoids, alkaloids, etc. have been identified as potential autophagy modulator and multidrug-resistance-reversal agents [22]. In addition, tunicamycin has been initially identified as a natural antibiotic and anticancer agent. It has suggested that tunicamycin inhibits N-glycosylation to aggravate endoplasmic reticulum stress, trigger autophagy, and increases the sensitivity of gastric cancer cells to Adriamycin and Vincristin. Moreover, the natural product genipin can induce p53 and DRAM expression and trigger apoptosis and autophagy in GC [22]. Out of all GC cases, 95% of them are adenocarcinomas, resulting in a multistep cancer progression (Correa Cascade) [23,24]. Based on the above, chronic gastritis followed by atrophic gastritis leads to intestinal gastric metaplasia, which further leads to dysplasia and adenocarcinoma [24]. There are two histological entities for GC—(i) the diffuse and (ii) the intestinal types of GC [25]—with the former being less differentiated than the latter, while the latter is well-differentiated with more frequent occurrence and a better outcome [26]. 

Gene mutations haveemerged in many inherited cancer syndromes, which lead to GC occurrence such as the loss of one allele of Cadherin 1(CDH1) gene, which normally encodes E-cadherin, an adhesion molecule [27,28], resulting in HDGC, a dominant autosomal syndrome that promotes not only cancer cell growth beneath the gastric epithelium but also colorectal cancer and extra-gastrointestinal malignancies [27,29]. Another mutation that is also found in HDGC is the one of Catenin Alpha 1(CTNNA1) gene, which also encodes an adhesion molecule, the alpha-E-catenin, taking part in the Wnt signaling pathway [27,30]. In GAPPS, the loss of an allele in 5q22 has been reported, as well as a point mutation in the promoter of the gene [29]. A predisposing mutation for Lynch syndrome is Glutathione S-Transferase Mu 1 (GSTM1)-null mutation, which is also correlated with interleukin gene expression mainly for IL-10 and IL17 [8,29]. Another hallmark mutation is the one of TP53 gene, the one most commonlyfound in GC (40% of GC cases), which is normally a crucial tumor suppressor, for deterring genomic instability. ΒRCA2 mutation has been associated with elongated survival [27,30], while the ARID1A mutant gene that is involved in chromatin remodeling is also found in GC cases, as well as the RHOA mutant gene [27]. Epigenetic aberrations are also reported, including the expression of non-coding RNA regulatory sequences [31] and hypermethylation in CpG islands [30]. More specifically, miR-21, a biomarker for GC diagnosis, has key role in epigenetic regulation, accounting for gastric cancer growth, cancer cell death, and invasive tumor behavior [31]. Other epigenetic modifications are that it suppresses expression of miR-15b,26a,145, as well as375 and 574 [32]. Of note, the combination chemotherapy regimens included mostly “older” regimens combination of 5- Fluorouracil/anthracyclines or platinum-based agents and therefore might have had optimal efficacy [27]. Thus, taking advantage of genomic alterations, new treatment strategies are taken into consideration, such as the expression of human epidermal growth factor receptor 2(HER2) and VEGR2, which constitute a drug target in GC, by using trastuzumab, positive HER2 tumors, and ramucirumab, respectively [26]. The multi-step procedure of autophagy could also be targeted in many steps such asPI3K, in cases of PIK3CA mutation, which is reported in 40% of hypermutated gastric malignant tumors. Moreover, EBV-positiveGC or MSI types of PD-1/PDL1 blockers could be used, due to the overexpression of PD-L1 in these tumors [27]. 

Based on a large number of preclinical studies, disturbances of autophagy machinery areclosely associated with tumorigenesis, as well as with metastasis and dismal outcomes, althoughit may act as a putative therapeutic approach for different cancer types, including gastric cancer. In this review, we gathered information from the current clinical and preclinical research data about autophagy modulation in gastric cancer and the therapeutic strategies for this highly invasive malignancy.

## 2. A Review of Autophagy

**Figure 1:**
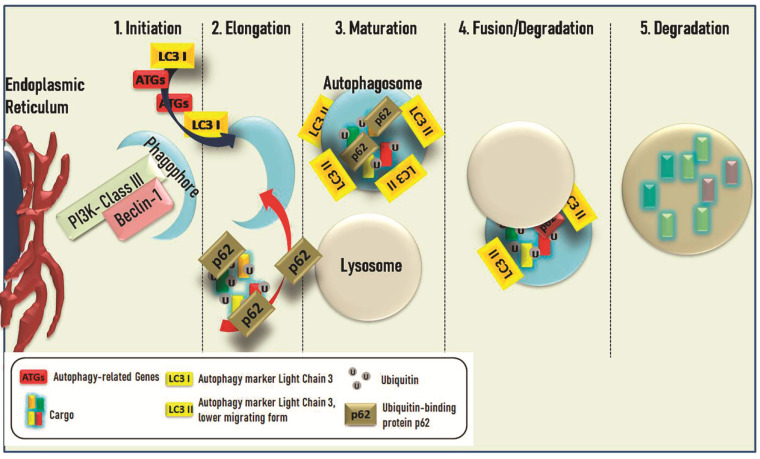
Schematic drawing showing the main steps of the autophagy process from phagophore to autophagosome formation.

Autophagy constitutes a highly, strictly regulated homeostatic mechanism, which is composed of multiple steps for the reassurance of the ideal conditions for cellular survival and protection [33]. This is succeeded byrecycling of over-matured and destructed organelles, which could be otherwise agglomerated and possibly minacious for cellular homeostasis [34]. As a mechanism is classified into macro-and micro-autophagy with the former, including the formation of phagophore for the isolation of cargoes and, later on, the fusion of the autophagosome with lysosome and the creation of autophagolysosome for their final degradation, the latter begins with the engulfment of the defected organelles via membrane invagination [35]. Chaperon-mediated autophagy (CMA) is a distinct entity, in which a chaperone identifies the proteins that will undergo destructionvia distinguishing a highly selective KFERQ (consensus pentapeptideof cytosolic chaperone hsc70)-motif in the over-matured or impaired proteins and delivers them in lysosomal membrane protein 2A (LAMP-2A) [36]. The first step of this procedure is (i) induction, including the inactivation of mammalian target of rapamycin (mTOR), under stressful conditions, followed by the activation of Unc-51-like kinase1 complex (ULK1) for the isolation of the cargoes [33]. In the second step, (ii) nucleation, class III PI3K is activated (phosphorylated) by ULK1, which is followed by the formation of the Beclin-1-PI3K complex [37,38]. The next step (iii) includes the membrane-elongation of the phagophore, resulting insubsequent conjugations of ATG5-ATG12 and ATG6-LC3I, with the latter LC3I to lipid phosphatidylethanolamine (PE), forming the LC3II (an insoluble form of LC3I). These conjugations promote further recruitment of cargoes and restrain the final products of their degradation [35,38]. The next step is the (iv) formation of autophagolysosome, leading to the last step (v), namely the degradation and recycling of the cargo [35]. The conditions in whichthis homeostatic mechanism is initiated, including hypoxia, lack of crucial nutrients, or inflammation [39], are extreme in order to ensure adequate energy for the cells. However, this mechanism has a dual role by be9jginvolved in carcinogenesis, as either a suppressor or promoter [40]. Research about the role of autophagy in GC and the possible use of autophagy-participating molecules have beenin the spotlight the recent years.Each autophagy stepis characterized by a large numberof proteins or structures that are putative targets for different agents, demonstrating them as possible biomarkers, prognostic factors, as well as drug targets. The main steps of the autophagy process are described in Figure 1. In this review, we will shed light on the importance of autophagy in gastric carcinoma and the opportunities for new therapeutic anti-cancer strategies.

Autophagy consists of several sequential steps. In order to form the main part of autophagy, autophagosome, several distinct morphological changes occur. Initiation (1) is the first step. This procedure consists in the formation of a double-membrane structure, the phagophore, after activation of PI3K-classIII–Beclin-1 complex in endoplasmic reticulum or other double-membrane organelles. Elongation (2) is the next step where the new-formed phagophore begins to enclose Ubiquitin-labeled cytosolic cargos. Several proteins such as LC3 (LC3-I is conjugated to phosphatidylethanolamine to form LC3-phosphatidylethanolamine conjugate or LC3-II, responsible for the autophagosomal membrane structure), Atgs (Autophagy-related genes), and p62 (an adaptor protein responsible for the docking of cargoes), which have a key role in the process. In the maturation (3) step, the Autophagosome has already formed following the fusion of Lysosome and Autophagosome in the fusion/degradation (4) step. In the new form structure, autolysosomes, where the degradation step (5) occurs and the cytocolic cargos are digested from lysosomal enzymes with the release of the products in the cytosol.

A large array of molecular machinery responsible for autophagy has already been identified. Specialized molecules, such as enzymes, kinases, and phosphatases, that can bind and hydrolyze guanosine triphosphate (GTPases) participate in the autophagy process, all encoded by autophagy-related (Atg) genes. The main ATGs, the autophagy step where they participate, and their molecular function are presented in Table 1.

## 3. Autophagy in Gastric Cancer

Autophagy is a crucial homeostatic mechanism for proper cellular function under extreme circumstances such as lack of energy, vital nutrients, and oxygen deprivation, which can create stressful conditions for cellular activity.However, its serves not only as a cellular conservation mechanism, acting as a suppressor for tumorigenesis via the recycling of defected organelles and proteins, but also as a tumor promoter of carcinogenesis, especially in later stages of the disease [41]. In addition, autophagy appears to also regulate tumor metastasis in gastric cancer.It is believed that autophagy operates as both pro- and anti-metastatic. The process of tumor metastasis is complex and depends on several events such as neo-angiogenesis, formation of tumor microenvironment, breakdown of extracellular matrix, and epithelial-to-mesenchymal transition (EMT). The role of autophagy in tumor metastasis is believed to be both pro-metastatic and anti-metastatic [41]. 

### 3.1. Regulation of Autophagy by MicroRNAs (miRNAs)

The interrelation of short non-coding RNA sequences of mRNA (miRNAs) with gastric carcinogenesis is in the spotlight in recent studies. These miRNAs are molecules composed of 19–22 nucleotides that contribute to the regulation of DNA expression, which are bound in 3′-UTR of mRNA sequence [42]. The deregulation of these molecules contributes to the progression of gastric malignancy, as well as its outcome, which makes them potent biomarkers for prognosis and diagnosis [42]. The impact of miRNAs in autophagy enhances the complexity of GC and it requires more investigation [43]. 

### 3.2. Regulation of Autophagy by miRNAs as Tumor Suppressor Genes

Many studies have reported that specimens with gastric malignancy present less expression of miR-1265 than normal tissues. MiR-1265 binds in the region of the gene that encodes calcium-binding protein 39 (CAB39), a key protein for the formation of CAB39- LKB1- STRAD complex, in the Thr172 site of their junction [44]. The CAB39–LKB1–STRAD complex normally influences the phosphorylation of the AMPK signaling pathway, which presents a 100-fold increase [45]. AMPK pathway is closely connected with an autophagic mechanism, which has a tumor promoter role in GC development. The role of miR-1265 is crucial, as it down-regulates the expression of CAB39, regulates the phosphorylation of AMPK, and inhibits the initiation of the autophagy pathway [46]. 

Another non-coding RNA is miR-495-3p, which contributes to the inhibition of autophagy-promoted gastric carcinogenesis, as well as to the conversion of multi-drug-resistant (MDR) cases of GC, via the downregulation of mTOR pathway, while the limited action of miR-495-3p enhances the malignant phenotype [44]. The same role, as down-regulators of the mTOR signaling pathway for autophagy are miR-21 [47], miR-361-5p [48], and miR-375 [44]. Decreased progression and multiplication of gastric cancer cell lines are associated with the expression of other miRNAs such as miR-532-3p, miR-181a, miR-133a-3p, miR-30a, and miR-1et-7a [44]. 

### 3.3. Regulation of Autophagy by miRNAs as Oncogenes

Autophagy pathway inhibition is also reported under the influence of miR-21 in the intracellular signaling pathway of the mTOR-PI3K/AKT axis, which has a close relation with diamminedichloroplatinum (DDP)-resistant gastric malignant cell lines [47]. Ultraviolet-radiation-resistance-associated gene (UVRAG) interrelates with Bcl-2, which normally leads to autophagy activation and finally to GC degradation. The binding of miR-183 in 3′-UTR of UVRAG inhibits the autophagy pathway and apoptosis of GC under conditions of nutrient deficiency, constituting miR-183 as an oncogene for autophagy mechanism [49]. However, the above condition opposes the potent role of miR-183 as a tumor suppressor mechanism in GC [50]. 

### 3.4. Regulation of Autophagy by Long Non-Coding RNAs (lncRNAs)

LncRNAs constitute longer non-coding RNA sequences (longer than 200 nucleotides), which closely influence the cell cycles, including at the level of transcription, as well as in the level of pre- and post-transcriptional processes, contributing not only to physiological mechanisms but also to non-physiological ones [51]. LncRNAs have an indirect regulatory role in the autophagy pathway via acquiring the role of competing for endogenous RNA (ceRNA) or via their direct adjustment to autophagy-related proteins that obtain a modified expression and functional role, while lncRNAs also contribute to acquisition of drug resistance by cell lines [22]. 

Many reports illustrate a relationship between the overexpression of RNA genes in chemo-resistant GC cases, such as the hepatocellular carcinoma up-regulated lncRNA (HULC), the HOXA distal transcript antisense RNA (HOTTIP), as well as the metastasis-associated lung adenocarcinoma transcript 1 (MALAT1) [52,53]. Based on a recent study [54], another potent function of HOTTIP is illustrated that obtains the role of ceRNA for miR-216a-5p, leading to the overexpression of BCL-2 and the downregulation of the Beclin-1 molecule. Other scientific reports have exhibited the interrelation of HOTTIP expression with autophagy activation and inhibition, with the former being when HOTTIP expression is oppressed, increasing DDP sensitive GC cell lines and the latter when it is up-regulated, andthis overexpression further leads to DDP-resistant GC cases [54]. 

According to a recent study [55], the inhibition of *ATG12* via the regulatory effect of miR-23b-3p is attenuated due to the effect of MALAT1 on miR-23b-3p, on whichMALAT1acts as a ceRNA molecule, resulting in the initiation of autophagy pathway and the generation of GC cell lines resistant to vincristine. Furthermore, the binding of MALAT1 on miR-30 up-regulates the *ATG5* expression, inducing autophagy mechanisms and creating gastric cancer cells resistant to DDP [56]. 

The contribution of HUCL to the induction of DDP-resistant gastric malignant cells, as well as autophagy processes, where HUCL impedes FoxM1 from being ubiquitinated while targeting HULC, can reduce DDP resistance and oppresses autophagy pathway [22]. 

Moreover, the latest studies showed the correlation between worrisome prognosis and HAGLROS expression, which are lncRNAs interrelated with the mTOR signaling pathway for autophagy. Overexpression of HAGLROS lncRNAs contributes to mTOR activation, leading to autophagy oppression and GC progression [57]. 

### 3.5. Regulation of Autophagy by PI3K/AKT/mTOR Signaling Pathway

Autophagy, as was discussed above, is highly regulated ateach step; one of these regulatory pathways is PI3K/AKT/mTOR axis, constituted by three distinct protein sub-molecules: (i) the PI3 kinase, (ii) the protein kinase–b (PKB/AKT, and (iii) the mTOR) [58]. This signaling pathway is crucial for the regulation of the autophagy pathway, which has an inhibitory effect, allowing cancer cell growth and progression [58]. In GC, this signaling pathway, when it is in action, promotes gastric cell proliferation, invasion, and durability and enhances the GC resistance to chemotherapeutical agents [59]. 

Protein kinase-B or AKT action is correlated with drug resistance, especially for antineoplastic agents such as cisplatin, 5-fluorouracil (5-FU), mitomycin C, and doxorubicin [60], via its stimulatory effect on mTOR, leading to the inactivation of autophagy and the accumulation of defective molecules, as well as tumor cell growth and multiplication [33]. Targeting PI3K/AKT/mTOR canbe used as a potent antineoplastic therapeutic method via the suppression of the pathway using flavonoids, which permits the inactivation of mTOR, the induction of autophagy, and the gastric tumor cell death by stopping them in the M/G2 cycle checkpoint [61]. Last but not least, inhibitory agent for PI3K/AKT/mTOR signaling pathway is attenuatedYWHAZ, which permits the autophagy process for human gastric cell lineBGC-823 [62]. 

### 3.6. Regulation of Autophagy by AMPK Signaling Pathway

A crucial signaling pathway for the initiation of autophagy is AMPK, which presents a variety of actions, including its regulatory effect on the MAPK/ERK pathway, the suppression of PI3K-AKT/mTOR pathway, and the phosphorylation of ULK1 complex in autophagy [63]. The antineoplastic action of the above pathway is also implied intissue specimens, where it appears lower in malignant tissues compared to physiological tissues, while AMPK suppression is closely linked with carcinogenesis [64]. The stimulatory effect on AMPK is exhibited by Peril aldehyde action, which succeeds via the phosphorylation in S428 and S307 positions in LKB1. Furthermore, Metadherinalso phosphorylates and activates AMPK, influences ATG5 and further inducts autophagy. However, it increases chemoresistance in gastric malignant cells, especially for antineoplastic agents such as etoposide, 5-FU, and paclitaxel, as well as doxorubicin and cisplatin [45].

### 3.7. Autophagy and Helicobacter pyloriin Gastric Cancer

Many studies have shown the relationship between the Gram (–) bacterial infection with *H. pylori* and gastric carcinogenesis. The virulence factors that *H. pylori* produces have a major contribution to GC development [65]. These factors include the cytotoxin-associated gene A (CagA) and the vacuolating cytotoxin (VacA) [66]. VacA has a key role in autophagy, as it takes part in the creation of autophagosome, while its induced damage is limited by the autophagy induction and VacA degradation [67]. There are many reports showing that the *H. pylori*-associated activation/initiation of autophagy is not only in gastric mucosal cells but also in macrophages [68]. However, there is a xenophagy mechanism, an autophagic pathway for pathogens like *H. pylori*, which leads to its elimination and degradation [69]. In addition, the genetic background of hosts makes them prone to *H. pylori* such as in cases of homologous individuals for atg16L1gene [70]. There is evidence that suppression ofATG12 expression allows the action of VacA in host cells [71]. Targeting the autophagy-related proteins and mTOR could be effective for the elimination of *H. pylori* from gastric epithelial cells such as the activators of mTOR, while suppression of autophagy with agents such as3-methyladenine promotes the *H. pylori* intracellular multiplication [72]. Based on what waspreviously reported, autophagy illustrates a protective mechanism against the toxic effect of virulence factors in the short term, while in the long term, the chronic existence of VacA in gastric epithelium, leading to reactive oxygen species (ROS) overproduction, autophagy-pathway deregulation, and p62/sequestosome 1 (SQSTM1) accretion, which result in disturbance of nuclear factor-κB (NF-κB) pathway and tumorigenesis [67]. ATG sequestosome 1 (SQSTM1)/p62, a ubiquitin-binding protein in autophagy in the exterior membrane of autophagosome [35], is considered one more potential prognostic factor, with bad prognostic value in GC, less lymphatic spreading, and less tumor differentiation [73]. 

### 3.8. Atgs in Tumorigenesis of Gastric Cancer

The initiation step includes a critical autophagy-related protein, mTOR, which is normally inhibited atthe beginning of the pathway [33]. However, when this protein is activated, macroautophagy is inhibited, which leads to the accumulation of toxins and impaired organelles, allowing the cancer cell to grow and survive [35]. 

There is a major involvement of Beclin-1 (ATG6 homolog in yeast), which has a key role in the induction of the autophagic pathway and the formation of the autophagosome. Overexpression of Beclin-1 is reported in malignant gastric tissue specimens [74], which is mainly cytoplasmic [58], as well as an increased expression of LC3I and its converted form ofLC3II (ATG8 homolog in yeast) [35]. Disturbances in Beclin-1 gene encoding are demonstrated in many malignant gastric cell lines, correlated with physiological gastric cell lines, such as GES -1 [67]. However, the occurrence of the Beclin-1 mutant gene is not common, as it accounts only for 2.8% of GC cases, according to Lee’s report [75]. Beclin-1 levels have prognostic value, with higher values found in cases with worrisome prognosis, while based on one study, the levels of this protein are slightly or not expressed in normal gastric tissues and increased in 50.9%of the cases with gastric malignancy [76]. Due to this fact, it could be used as a prospective cancer prognostic biomarker inGC. Despite the fact that Beclin-1 is related to a worse prognosis, it is not related to lymphatic metastasis or distant-organ metastasis [77]. 

The same happens for another autophagy-related protein mutation, that of ATG5, which is presented only in 1.5% of GC cases and 21% of them and where there Atg5 expression is absent [78]. Increased or attenuated expression of many ATGs is closely related to gastric carcinogenesis and the disturbances of GC cells’ apoptosis. In many reports, such as the one of Vigen et al. for gastric adenocarcinomas, there is an increased expression of ATG16 (80% of GC cases) and ATG5 (80% of GC cases), while in cases of gastric carcinoid, the expression of ATG16 is higher (90%) in comparison with that of ATG5 (60%) [79]. 

GC cases with high MSI are associated with cases of GC, where Atgs mutations exist, such as in the case ofatg9B, atg2B, atg12, and ATG5, which account for 28.1% of the cases, but there are none described in GC with low MSI [67]. Last but not least, ultraviolet-radiation-resistance-associated gene (UVRAG), binding to Beclin-1, has a crucial role in the induction of the autophagy pathway, and it is also correlated with high MSI GC (9.4%), aphenomenon that reveals a possible disturbance in autophagy mechanism in high-MSI gastric malignancies [80]. Further investigation about the complex role of ATG could answer many questions about their oncogenic role in GC.

## 4. Targeted Autophagy as Putative Therapeutic Approach

Based on the qualities of autophagy as either a suppressor or stimulator of cancer growth, autophagy-based anticancer drugs are in the spotlight, including autophagy inhibitors and inducers. Autophagy inducers, such as mTOR inhibitors in cases of GC-disseminated-type or AMPK homeostatic pathway activators such as the antibiotic substance Tigecycline [35], could be used in cases of chemoresistant GC, in which other anti-cancer treatments failed to reduce the cancer progression. These are PI3K complex inhibitors and lysosome-specific targeted drugs, such as hydroxychloroquine (HCQ) and chloroquine (CQ) [81]. Lysosomes could be used as a therapeutic target via the blockage of the formation of autophagolysosome [81,82]. PI3K inhibitor and CQ could have a synergic role with other types of anti-cancer treatment, such as cisplatin, which reduces the chemoresistance of gastric cancer lines [83], andin case of its combination with oxaliplatin, they have enhanced anti-growth action for gastric cancer cells.

### 4.1. Autophagy Enhancer Agents

Numerous scientific research studies indicate the close relationship between the tumor micro-environment with autophagy pathway, as well as with the inducted anti-neoplastic immune reaction, in many malignancies, including GC.The influential characteristics of autophagy open up new horizons for the evolution of new anti-cancer substances.Some of the most remarkable autophagy inductors are Rapamycin inductors, including the inhibitors of mTOR, rapalogs, and Rapamycin analogs [84]. Some noteworthy rapalogs are everolimus, as well as temsirolimus, while deforolimus is a rapamycin analog, which activates the autophagy mechanism [84]. It is reported that the addition of Paclitaxel inEverolimus therapy has a significant suppressive effect on endometrial cancer cell progression [85]. There is a notable effect of Rapamycin as an anti-cancer treatment, which includes the activation of the autophagy pathway, the enhancement of radiationtherapy’s effect on lung cancer cells of the -A549 type, and it also influences the DNA- repair process [86]. Although these autophagy inductors have a significant potential role in anti-neoplastic therapeutic schemes, further investigation is needed for their usage in clinical oncology [87]. 

Moreover, Metformin, a noteworthy substance for its pharmaceutical properties, constitutes an autophagy activator [88], such as in the case of pulmonary adenocarcinoma, which undergoes apoptosis through tumor-necrosis-factor (TNF-related-Apoptosis-Inducing-Ligand (TRAIL)) [89]. For breast malignancy, in the absence of mutant BRCA1 gene, metformin can be included in therapeutic schemes with spautin-1, which constitutes an autophagy suppressor, resulting in an altered mitochondrial functional state and inducing a notable reduction incancer cell survival and progression [88,90]. Furthermore, significant autophagy suppressors are mTOR inhibitors, such as alkaloids [90,91], including cepharanthine, liensinine, andisoliensinine [85], while they induce phosphorylation of the AMPK pathway. The above autophagy activators demonstrate great results in cases of resistant apoptosis in Mouse Embryonic Fibroblasts (MEFs) [92]. Another autophagy activator, a pan-inhibitor of anti-apoptotic Bcl-2 proteins that exhibits a cytotoxic effect on cancer cells through both apoptosis-dependent and -independent pathways, the so-called Obatoclax [93], is correlated with mitochondrial-pathway apoptosis via targeting the Bcl-2 protein family, and it is also linked with autophagy-complexes’ death via necroptosis [88,92]. Last but not least, the antioxidant omega-3polyunsaturated fatty acids have a key role in autophagy activation [94], constitute a potent adjuvant anti-cancer agent, such as in case of cholangiocarcinoma, while they do not have notable toxicity [85]. These agents activate 15-hydroxyprostaglandin dehydrogenase, which leads to the suppression of prostaglandin E2 (PGE2), which is a causative factor for the above malignancy [88]. In Table 2, we summarize some of the autophagy activators and the main mechanisms of action that are mostly known.

### 4.2. Autophagy Inhibitors

In the past few years, except for the conventional cancer therapies such as radiation therapy and chemo-immunotherapy, a new anti-cancer therapeutic strategy is in the spotlight, including autophagy-based treatments, such as autophagy inhibitors [95]. As was previously underlined, autophagy can serve as either a suppressor or promoter of carcinogenesis. These new regimens make use of the basic properties of the autophagy pathway and their influence on the metabolic state and the endurance of cancer cells [34]. Autophagy inhibitors that are broadly noted are HCQ, CQ, and Lys05 (dimeric of CQ), which are used in many cancers, interfering with the formation of the autophagolysosome. The latter exhibits a strong anti-neoplastic effect as a modifier of lysosomal function [96]. Despite the fact that they exhibit adequate effectiveness as a combination treatment with other anti-cancer regimens [97], as a monotherapy, they demonstrate a restricted performance as a consequence of their discontinuous inhibitory effect [98]. In animal models, the combination of CQ with Interleukin-2 has shown benefits in secondary hepatic cancer, with limited toxic effects and improved prognosis [95]. A great improvement in pancreatic cancer progression is also noted, in which Gemcitabine is combined with HCQ, with an important decrease inCA19-9 neoplastic marker (60%) [99]. Although these inhibitors show beneficial effects on cancer treatment, they can provoke interactions with other pharmaceutical agents, and they can induce alterations in the tumor microenvironment [96,98]. 

Due to the fact that their effect cannot be assessed by specific markers, other current inhibitors are used in therapeutic schemes [95]. The initiation step is highly regulated by many proteins such as the ULK1 as well as the Vps34-signaling pathway, including some critical proteins such as Vps34, Beclin-1 and Vps18, which have a significant role in the conveyance of the vesicles, as well as the lysosomes [100]. Inhibition of the above key-proteins for the initiationstep of autophagy exhibits an intense anti-neoplastic effect, starting with SBI-0206965, a highly selective ULK1 inhibitor [101], as well as Beclin-1 suppressors, which induce cancer cell death via the stimulation of more CCL5 expression in cancer cells that attract Natural-Killer cells to them [95]. Moreover, suppressors such as SAR405 inhibit Vp34 and lead to the alteration of lysosomal function [100], while spautin -1 inhibits USP10 and USP13 peptidases (ubiquitin-specific peptidases) [102]. Additionally, the level of autophagolysosome formation is targeted by many medical substances such as clomipramine, desmethylclomipramine (DCMI), and [103], with the enhancement of DCMI efficiency by adding doxorubicin, as was demonstrated in in vitro studies [104]. 

In some cases, inhibition of the autophagy pathway could limit the immune response to carcinogenesis and could lead to cancer cell progression and survival.However, this hypothesis has proved wrong based on studies for breast cancer and melanoma. Subsequently, for the intensification of the anti-neoplastic immune response, autophagy suppressors are used in combination with other chemotherapeutic substances [35,105]. In Table 3, we summarize some of the autophagy inhibitorsand the main mechanism of action that are mostly known.

Finally, the utilization of autophagy properties opened new horizons for developing new anti-cancer therapeutic agents and intensifying the effect of other conventional anti-neoplastic treatments for many malignancies.For example, the inactivation of AKT can succeed via Perifosine, which constitutes an alkylphospholipid that demonstrates anti-cancer activity. Combinational treatment with Perifosine and NH4Cl or CQ induces apoptosis, as well as limitation of tumor progression and expansion [106]. It is reported that a combinational therapeutic scheme with HCQ, an autophagy inactivator, and Temsirolimus, which is an mToR inactivator, has been utilized in late-stage solid tumors or in case of melanoma; however, this clinical trial is in phase I [107]. Moreover, in head and neck malignancies, such as in squamous cell carcinoma, the use of CQ with either oprozomib or carfilzomib, which are next-generation proteasome inhibitors, demonstrates activation of autophagy pathway and cancer cell destruction [108]. Another combinational therapy is propachlor with the mTOR inhibitor Everolimus, which act as autophagy activators and lead to malignant celldeath in prostate cancer. Another mTOR inhibitor, RAPA, when combined with temozolomide-treated, shows beneficial effects in cases of glioma, with the death of U251 cells [109]. Autophagy induction via isoliquiritigenin, in combination with 3-MA, leads to the enhancement of anti-cancer response in ES-2 cells [109]. Multiple events, such as the accumulation of proteins induced by CQ in lysosomes and protein aggregation in cytosol, induced by Bortezomib, possibly leads to mitochondrial function disturbances, followed by the activation of Apaf-1, which contains apoptotic complex and the release of cytochrome c [109]. Further research is needed for the handling of GC, which remains a difficult task in clinical practice.

## 5. Conclusions

Overall, management of GC remains a difficult task for clinical practitioners, mainlyattributed to the increased chemoresistance of this malignancy in conventional therapeutic approaches. The autophagypathway is the focus of many scientific studies with respect toits properties as a physiological cellular adaptation mechanism in stressful conditions, as well asits binary function in cancer, either as suppressor or inducer of cancer progression. Promisingopportunities have opened up for the development of new therapeutic strategies againstmany malignancies, including GC. The combination of conventional andautophagy-based anti-neoplastic agents are showing promising results in in vitro studies. However, there are limitations such as discontinuous inhibition of autophagy, interactions with other pharmaceutical agents, and alterations in tumor microenvironment and the anti-neoplastic immune response.Despite the abovelimitations, autophagymodulators open up new horizons as treatmentstrategies for GC, as well as a combinational treatment with other chemotherapeutic agents, promising better therapeutic results and elongated survival by enhancing chemosensitivity or restoring the drugresistance of GC.In conclusion, further investigation is required for the controversial role of autophagyand the manipulation of its multiphasic nature, with a wide variety of druggable targets, for the creation of a novel anti-neoplastic medical treatment.

## Figures and Tables

**Table 1:** Yeast and human autophagy-related genes.

ATGs	Human Orthologue	Autophagy Step	Molecular Function
Atg1	ULK1/2	Induction	Kinase
Atg2	ATG2A, ATG2B	Nucleation	Protein binding
Atg3	ATG3	Elongation	Ubiquitin-like ligase
Atg4a	ATG4A, ATG4B	Elongation	Cysteine-type endopeptidase
Atg4b	ATG4C	Elongation	Cysteine-type endopeptidase
Atg5	ATG5	Maturation	Ubiquitin-like ligase
Atg6	BECN1	Nucleation	Kinase
Atg7	ATG7	Elongation	Ubiquitin-activating enzyme
Atg8a	GABARAP	Elongation	Ubiquitin-like
Atg8b	MAP1LC3C, MAP1LC3B2	Elongation	Ubiquitin-like modifying enzyme
Atg9	ATG9A, ATG9B	Nucleation	Protein binding
Atg10	ATG10	Maturation	Ubiquitin-like ligase
Atg12	ATG12	Maturation	Ubiquitin-like
Atg13	ATG13	Induction	Protein kinase binding
Atg14	ATG14	Nucleation	Kinase
Atg16	ATG16L1, ATG16L2	Maturation	Ubiquitin-like ligase
Atg17	RB1CC1	Induction	Protein kinase binding
Atg18a	WIPI2	Nucleation	PIP2 binding
Atg101	ATG101	Induction	Protein binding

Atg: Autophagy-related genes; ULK: Unc-51-Like Autophagy Activating Kinase 1; BECN1: Beclin-1.

**Table 2:** Autophagy activators and their main mechanism of action.

Agents	Mechanism of Action	Target
**Rapamycin**	mTORC1 inhibitor	Formation of Autophagosome
**Deforolimus**	mTORC1 inhibitor	Formation of Autophagosome
**Temsirolimus**	mTORC1 inhibitor	Formation of Autophagosome
**Everolimus**	mTORC1 inhibitor	Formation of Autophagosome
**GDC-0941**	PI3K Class I inhibitor	Formation of Autophagosome
**GDC-0980**	PI3K and mTORC1 inhibitor	Formation of Autophagosome
**Tat–Beclin-1 peptide**	Releases Beclin-1 into cytoplasm	Formation of Autophagosome
**Perifosine**	AKT inhibitior	Formation of Autophagosome
**Metformin**	AMPK activator	Formation of Autophagosome
**fluspirilene**	Antagonists of L-type Ca^2+^ channels	Lysosome
**cepharanthine**	Natural alkaloid	Autophagic flux
**isoliensinine**	Natural alkaloid	Autophagic flux

mTORC1: mammalian target of rapamycin complex 1; AMPK: 5′ AMP-activated protein kinase; PI3K: phosphatidylinositol 3-kinases; AKT: Protein kinase B (PKB); Beclin-1: the mammalian ortholog of the yeast autophagy-related gene 6 (Atg6).

**Table 3:** Autophagy inhibitors and their main mechanism of action.

Agents	Mechanism of Action	Target
Chloroquine (CQ)	Neutralizes the acidic pH of intracellular vesicles	Lysosome
Hydroxy-chloroquine (HCQ)	CQ derivative	Lysosome
Bafilomycin A1	Inhibition of lysosomal acidification	Lysosome
Azithromycin	Inhibition of lysosomal acidification	Lysosome
Concanamycin A	Inhibition of lysosomal acidification	Lysosome
3-Methyladenine (3-MA)	PI3K- Class III inhibitor	Formation of Autophagosome
Wortmannin	PI3K- Class III inhibitor	Formation of Autophagosome
LY294002	PI3K- Class III inhibitor	Formation of Autophagosome
LY3023414	PI3K- Class III inhibitor	Formation of Autophagosome
SAR405	Vps18 and Vps34) inhibitor	Formation of Autophagosome
SB203580	Inhibit trafficking of Atg9	Formation of Autophagosome
Paclitaxel	Microtubule stabilizer inhbits phosphorylation of VPS34	Formation of Autophagosome
SAHA	Inhibit fusion of autophagosome and lysosome	Formation of Autophagosome
Sputin-1	(USP10) and (USP13) inhibitor	Formation of Autophagosome
NSC185058	ATG4 inhibitor	Formation of Autophagosome
Verteporfin	Alter lysosomes accedification	Formation of Autophagosome

VPS34: vacuolar protein sorting-associated protein 34; mTORC1: mammalian target of rapamycin complex 1; PI3K- Class III: Phosphoinositide 3-kinases (PI3Ks) class III.

## Data Availability

Data sharing not applicable. No new data were created or analyzed in this study. Data sharing is not applicable to this article.

## Abbreviations

ATGs	Autophagy-related genes
CAB	calcium-binding protein
CagA	cytotoxin-associated gene A
ceRNA	competing for endogenous RNA
CDH1	Cadherin 1
CMA	Chaperon-mediated autophagy
CTNNA1	Catenin Alpha 1
CQ	Chloroquine
DCMI	desmethylclomipramine
DDP	diamminedichloroplatinum
EAC	Esophageal adenocarcinoma
EBV	Epstein–Barr virus
EMT	epithelial-to-mesenchymal transition
FAP	Familial Adenomatous Polyposis
GAPPS	stomach syndrome
GC	Gastric cancer
GIM	gastric intestinal metaplasia
GSTM1	Glutathione S-Transferase Mu 1
HCQ	hydroxychloroquine
HDGC	Diffuse Gastric Cancer
HNPCC	Hereditary Non-Polyposis Colorectal Cancer
HOTTIP	HOXA distal transcript antisense RNA
*H. pylori*	*Helicobacter pylori*
IL	interleukin
KFERQ	consensus pentapeptide of cytosolic chaperone hsc70
LAMP-2A	lysosomal membrane protein 2A
lncRNAs	Long Non-Coding RNAs
MALAT1	metastasis-associated lung adenocarcinoma transcript 1
MEFs	Mouse Embryonic Fibroblasts
MSI	microsatellite instability
mTOR	mammalian target of rapamycin
PE	phosphatidylethanolamine
PGE2prostaglandin	E2
PPIs	Proton-pump inhibitors
UVRAG	Ultraviolet radiation resistance-associated gene
VacA	vacuolating cytotoxin
VEGF	Vascular endothelial growth factor
5-FU	5-fluorouracil
